# Neuromodulation Therapy in Heart Failure: Combined Use of Drugs and Devices

**DOI:** 10.19102/icrm.2020.110705

**Published:** 2020-07-15

**Authors:** Christopher O. Sobowale, Yuichi Hori, Olujimi A. Ajijola

**Affiliations:** ^1^UCLA Cardiac Arrhythmia Center, UCLA Neurocardiology Research Center, David Geffen School of Medicine at UCLA, Los Angeles, CA, USA; ^2^Department of Cardiology, Dokkyo Medical University Saitama Medical Center, Saitama, Japan

**Keywords:** Autonomic nervous system, heart failure, neuromodulation

## Abstract

Heart failure (HF) is the fastest-growing cardiovascular disease globally. The autonomic nervous system plays an important role in the regulation and homeostasis of cardiac function but, once there is HF, it takes on a detrimental role in cardiac function that makes it a rational target. In this review, we cover the remodeling of the autonomic nervous system in HF and the latest treatments available targeting it.

## Introduction

Heart failure (HF) is the fastest growing cardiovascular disease globally, estimated to affect more than 37.7 million people.^1^ Projections in the United States (US) suggest that the prevalence of HF will increase by 46% from 2012 to 2030, resulting in more than eight million people over the age of 18 years having HF.^2,3^ This growing prevalence is due to improved HF survival despite decreasing incidence, although some data suggest that improvements in survival could level off over time.^1^ Currently, the lifetime risk of HF is high (20%–45%) and HF remains the leading cause of hospitalization^4^ and 30-day readmission rates^5^ in the US. Despite the significant efforts and progress made in treating HF, the mortality rate remains high, with one in eight deaths linked to HF.^2^ Advanced therapies have been introduced to alleviate the broadening of HF such as left ventricular assist devices and cardiac transplantation but, given the limited available resources, more therapeutic targets need to be identified. The autonomic nervous system plays an important role in the regulation and homeostasis of cardiac function but, once there is HF, it takes on a detrimental role in cardiac function that makes it a rational target. In this review, we discuss the remodeling of the autonomic nervous system in HF and the latest treatments available that target it.

## The autonomic nervous system in heart failure

Cardiac function is determined by the complex interactions of the sympathetic (SNS) and parasympathetic (PNS) nervous systems together with regional responses and feedback from the central nervous system.^6^ Excitation of the SNS promotes norepinephrine (NE) release from the nerve endings, while the adrenal glands and medulla release NE and epinephrine. These catecholamines act on the adrenergic receptors (ARs), which subdivide into further subtypes (α1, α2, β1, β2, and β3).^7^ In the human heart β-ARs constitute about 90% of ARs and α1-ARs constitute about 10%.^8^

In acute HF, activation of the SNS is one of the first adaptations related to an increased release and decreased reuptake of NE induced by reduced stroke volume.^6,8,9^ NE and other neuromodulators support the maintenance of cardiac output by increasing the heart rate (HR) and contractility. In addition, NE also facilitates enhanced venous tone and systemic vasoconstriction to maintain blood pressure (BP). In contrast, chronic sympathetic stimulation in HF patients has detrimental effects. Persistently elevated NE levels increase cardiac stress via tachycardia, increased afterload, and high oxygen consumption, hastening the progression of ventricular remodeling. Simultaneously, elevated catecholamines bind with cardiac β-receptors and trigger G-protein-coupled receptor kinase upregulation inside cardiomyocytes, causing the downregulation and desensitization of β1 receptors at the plasma membrane.^5,6,9^ These reactions are considered to form a protective mechanism by which the heart is defended against excessive catecholaminergic toxicity, which has been found to cause cyclic adenosine monophosphate–mediated calcium overload, which can lead to cardiac cell death.^6,8,9^ Overexpression of β1 adrenergic receptors in mice resulted in early hypertrophy, interstitial fibrosis, and the upregulation of proapoptotic proteins, increasing the rate of apoptosis.^10^

PNS control of the heart is mediated by nicotinic acetylcholine receptors (nAChR) and muscarinic acetylcholine receptors (mAChR) via neurotransmitter acetylcholine (ACh).^6,11^ Vagal efferent signaling originates from the caudal ventrolateral medulla and extends to the ganglionated plexi on the atria, activating cardiac nAChR on the postganglionic neuron.^11^ The postganglionic fibers then reach the mAChR located on the myocardium and the situation progresses to PNS activation; in particular, it decreases the HR with a less significant reduction in contractility.^6,11^ The muscarinic receptor family consists of five subtypes (M_1–5_) and M_2_ receptors in particular, located at cardiac nerve endings, are essential for the physiologic control of cardiac function.^12^

The main physiological findings of PNS dysfunction in HF encompass the abnormal control of HR.^13,14^ Specifically, increased HR and decreased HR variability both correlate with increased mortality in HF.^6,11^ Regarding the mechanism at play, the stimulation of preganglionic vagal fibers was performed in HF dogs and controls and the HF group demonstrated a significantly lower response rate, confirming that functional abnormality was likely to be identifiable in the efferent limb.^15^ In addition, Vatner et al. demonstrated that, in a pacing-induced HF dog model, end-organ mAChR was upregulated, while downstream G-protein signaling was intact.^16^ Accordingly, dysfunction of nAChR is considered to be a strong candidate for abnormal vagal tone in HF. The abnormality of HR can be observed from early in the development of left ventricular (LV) dysfunction and how this process can be reversed is still unclear.

## Pharmacological therapy of heart failure: neurohormonal modulation

Pharmacological medications of HF are fundamental for patient wellbeing and are well-established. In particular, the importance of neurohormonal modulation in HF is evident given that the guidelines-directed medications available for HF—which include β-blockers, angiotensin system blockers such as angiotensin-converting-enzyme inhibitors (ACEIs) and angiotensin receptor blockers (ARBs), and mineralocorticoid receptor antagonists—all mainly work via the nervous system.

### β-blockers

β-blockers in HF inhibit the effects of long-lasting excessive NE levels on β-ARs due to the activated SNS. β-blockers contribute to the restoration of the downregulated β-ARs due to HF and, as a consequence, lead to an improvement in LV function.^17–19^ Interestingly, carvedilol (a mixed α1- and β-blocker) does not restore the number of β-ARs but does recouple the existing receptors and improves the signaling pathway efficiency.^20^ Long-term studies have shown β-blockers to reverse remodeling by decreasing LV mass and to revise ventricular shape to a more normal, elliptical appearance. β-blockers also inhibit pacemaker currents such as I_f_ to reduce the HR, which lessens the calcium entry into the failing myocytes to decrease cytosolic calcium overload.^21,22^ β-blockers also inhibit the phosphorylation of the cardiac ryanodine receptor at the sarcoplasmic reticulum that occurs in patients with HF. As a result, there is increased calcium recycling by the sarcoplasmic reticulum that promotes increased ejection fraction (EF).^23^

### Renin–angiotensin–aldosterone system inhibitors

In HF, the renin–angiotensin–aldosterone system (RAAS) is overactivated due to low renal perfusion and RAAS inhibitors such as ACEIs and ARBs play an important role in this pathological condition. As known from prior large-scale clinical trials, there is conclusive evidence that RASS inhibitors cause improvements in LV contraction, admission rates, death, and many other factors.^24–26^ The major cardiovascular effects of angiotensin II are mediated by angiotensin II type 1 receptors (AT1 receptors), which can be found in the vascular, heart, kidney, adrenal glands, and central nervous systems.^27^ Considering the relationship with the autonomic nervous system, the activation of AT1 receptors in the paraventricular nucleus and rostral ventrolateral medulla is reported to increase the activity of the SNS.^27,28^ In addition, the sympatho-inhibitory effects of AT1 receptor blockade, an ACE inhibitor (lisinopril), and of an AT1 receptor antagonist (valsartan) were examined in HF patients.^29^ Both lisinopril and valsartan significantly reduced plasma NE levels, although the reduction was significantly larger with valsartan than lisinopril (27% versus 6%; p < 0.05). However, no significant differences were observed in the effects on LV function, arterial pressure, aldosterone plasma level, or autonomic control of HR. In general, no significant difference has been observed between ARBs and ACEIs in reducing total mortality or morbidity rates.^25^ The mechanisms of regulating RAAS activity in the central nervous system and their contribution to HF treatment remain unclear.

### Mineralocorticoid receptor blockers

Mineralocorticoid receptor (MR) blockers (MRBs) inhibit the effects of aldosterone and cortisol. In HF patients, MRBs help to alleviate sodium-mediated fluid retention and vasoconstriction. The speculated mechanism is that aldosterone is co-expressed with the enzyme 11β-hydroxysteroid dehydrogenase II (11β-HSC2) and cortisol is converted to cortisone, which cannot activate the MRs.^30,31^ However, in HF patients, 11β-HSC2 may be downregulated, causing cortisol to activate the MR. The activated MR is correlated with the generation of reactive oxygen species, increased renal mesangial fibrosis, endothelial dysfunction, myocardial fibrosis, and myocyte death.^30^ Spironolactone has also been found to decrease the release of cardiac NE^32^ and improve cardiac 123I-MIBG (an NE analog) scintigraphic findings.^33^

### Angiotensin receptor–neprilysin inhibitors

Valsartan and sacubitril are part of a relatively new class of drugs referred to as angiotensin receptor–neprilysin inhibitors (ARNIs).^34,35^ Natriuretic peptides, degraded by neprilysin, have been found to increase renal perfusion, inhibit sodium reabsorption, and suppress RAAS and sympathetic activation. Neprilysin also increases the conversion of angiotensin I to angiotensin II, constituting the reason for why it is combined with the ARB valsartan. As HF progresses, the responsiveness to natriuretic peptides decreases, so the usage of ARNIs decreases RAAS activation and encourages diuresis. In the Angiotensin–Neprilysin Inhibition Versus Enalapril in HF (PARADIGM-HF) trial, ARNI therapy was compared with enalapril and found to achieve a 20% relative risk reduction in cardiovascular death and HF hospitalization.^36^

## Neuromodulation therapies have success in humans

Despite the remarkable progress made in pharmacological therapy for HF as shown by clinical trials, the morbidity and mortality rates of HF remain high. Recently, successful human studies of neuromodulation therapies that directly target the autonomic nervous system have been reported. We provide an overview of these studies in the following section of this review.

### Cardiac sympathetic denervation

In HF patients, excessive activation of the SNS plays an important role in cardiac remodeling and the occurrence of lethal arrhythmias. Cardiac sympathetic denervation (CSD) has primarily been adopted as an antiarrhythmic therapy for patients with severe ventricular arrhythmias. It is performed by surgically removing the lower half of the stellate ganglia through the T2–T4 thoracic ganglia^37,38^ and eliminates both afferent signaling and sympathetic postganglionic efferent to the heart. The beneficial effects of left-CSD (LCSD) have been well-investigated for the suppression of ventricular arrhythmias in long QT syndrome and catecholaminergic polymorphic ventricular tachycardia.^39–43^ Recently, the utility of CSD in refractory ventricular arrhythmia was shown in an international retrospective study assessing a total of 121 cases.^40^ CSD reduced 88% of the burden of ICD shocks (from 18 ± 30 to 2.0 ± 4.3 times per year) at a median follow-up of 1.1 years (p < 0.01).^40^

The efficacy of LCSD in HF therapy has also been considered. Conceição-Souza et al. conducted a pilot study where 15 symptomatic HF patients (LVEF ≤ 40%) were randomized to an LCSD group (n = 10) or non-LCSD group (n = 5).^44^ All subjected patients were continued on optimal medical therapy during the study. Six months later, LCSD had suppressed the progression of HF and improvements were observed in LVEF (from 25% ± 6.6% to 33% ± 5.2%; p = 0.03) and six-minute walking test outcomes (from 167 ± 35 m to 198 ± 47 m; p = 0.02). Currently, a prospective randomized trial of LCSD is being performed.^45^

### Renal nerve denervation

Renal nerve denervation (RND) achieves an effect by eliminating the renal sensory afferent and sympathetic efferent nerve fibers.^46^ This has been accomplished by surgically stripping the renal nerves or noninvasively treating by renal artery ablation. Elevated sympathetic nerve activity conducts through the renal efferent and causes NE spillover at the nerve endings. Consequently, α-AR activation leads to renal vasoconstriction.^47,48^ The NE level is reported to correlate with the development of HF. In addition, NE spillover in the heart is observed beginning in the early stage of HF, while its correlation in the kidney is more precise in severe HF.^49–51^ Simultaneously, vasoconstriction increases renal sodium retention and renin release, consequently raising the aldosterone level.^51^ High aldosterone levels provoke cardiac remodeling,^52^ influencing both morbidity and mortality. The afferent renal sympathetic nerves also contribute to the adjustment of BP largely through the actions of mechanoreceptors and chemoreceptors located in the kidney^46^ and, consequently, by modulating central SNS activity.

The initial feasibility of RND was as a treatment for refractory hypertension. Several trials were completed but no significant effects were shown when targeting patients with severe hypertension.^53,54^ On the other hand, in recent studies, RND has been reported to reduce BP when targeting patients with moderate hypertension (systolic BP ≥ 140 mmHg or < 170 mmHg).^55^ In addition, the utility of RND in both atrial and ventricular arrhythmias.^56–59^

The utility of RDN in HF patients was first reported in the Rehabilitation Enhancing Aging Through Connected Health (REACH) Pilot study.^60^ Patients with chronic symptomatic HF on optimal medical therapy underwent RDN. During six months of follow-up, RDN was associated with improvements in both symptoms and exercise capacity. No significant decrease in BP nor a worsening of renal function was observed and some patients were able to reduce their use of diuretic drugs. Consequently, more trials to prove the beneficial effects of RDN in HF patients were published. The Renal Denervation in Patients with Chronic HF and Renal Impairment Clinical Trial (SYMPLICITY-HF) was designed as a prospective randomized multicenter study including HF patients (n = 39) with LVEFs of less than 40%, New York Heart Association (NYHA) functional classes II to III, and receiving optimal medical therapy.^61^ Unfortunately, no significant improvement was shown in cardiac functions and symptomatic findings at 12 months after RDN. In this study, the ablated lesions were distributed only in the main renal artery from the ostium to the first major bifurcation and no treatments were applied distal to the main bifurcation of the renal artery, which may have been insufficient for RDN. Recent studies have found that targeting the renal artery branches or distal segment of the main renal artery resulted in markedly less variability of response than as seen with the conventional approach of only ablating the main renal artery.^62,63^ On the other hand, there was a 47% decrease in renal NE spillover and a 26% decrease in cardiac NE spillover at six months. Given the fact that renal spillover of NE is activated in HF patients and is predictive of mortality,^48,49^ it is still conceivable that RND might have a positive impact on HF symptoms and outcomes. In addition, more than 90% of patients were taking ACEIs/ARBs and β-blockers for optimal medical therapy; accordingly, the study may have not had an appropriate design or the numbers may not have been enough to accurately detect the effects of RDN.

Recently, a different pilot study investigating the effect of RDN in HF was reported.^64^ A total of 60 symptomatic HF patients (LVEF ≤ 40%, NYHA classes II–IV, and optimal medical therapy) were subjected and randomized to an RDN group and a medical therapy–only group. During six months of follow-up, the RDN group showed a salutary effect, with significant improvements observed in cardiac function, physical findings, and N-terminal (NT) prohormone B-type natriuretic peptide (pro-BNP) level. In this study, an irrigated catheter was used to obtain wide lesions and the physical conditions were also different. Further, the body mass index (BMI) of the patients included was 24.2 ± 2.8 kg/m^2^, while that for SYMPLICITY-HF patients was 31 ± 6 kg/m^2^, which may have influenced the distribution of the eliminated nerve fibers locating around renal artery.^64^ A reliable method to assess the completeness of RND still needs to be established to conclude the reactive nitrogen species effect in HF patients.

### Vagus nerve stimulation

The major goal of vagus nerve (VN) stimulation (VNS) in HF is to increase the parasympathetic tone. The beneficial effect of VNS in cardiac function has been reported in several animal studies. Vanoli et al. demonstrated that VNS prevents the occurrence of ventricular fibrillation during acute myocardial ischemia in dogs.^65^ Meanwhile, when VNS was performed in a pacing-induced HF canine model, cardiac function was improved and levels of inflammatory markers and the neurohormone, which suggests excitation of the SNS, were decreased.^66^ To summarize, VNS suppressed ventricular arrhythmia, improved survival and cardiac function, and reduced inflammation in these HF models.

VNS devices have initially been used for the treatment of epilepsy and refractory depression. Schwartz et al. reported the first pilot study to assess the feasibility and safety of vagal stimulation in patients with advanced HF.^67^ With six months of treatment, VNS reduced the LV volume and was able to improve physical findings such as NYHA class and six-minute walking test outcomes. Recently, three randomized trials were performed to assess the utility of a VNS device in HF patients. The Autonomic Neural Regulation Therapy to Enhance Myocardial Function in HF (ANTHEM-HF) trial was able to show a beneficial effect of VNS, while the Increase of Vagal Tone in Chronic HF (INOVATE-HF) and Neural Cardiac Therapy for HF (NECTAR-HF) trials revealed no significant improvement in HF.^68–71^ It is worth noting that each of these trials used a different stimulation protocol and titration of output. In the ANTHEM-HF trial, the titration of output was based upon the confirmation of autonomic engagement using changes in HR,^68^ while, in the other two studies, no significant reduction was observed in HR. Establishment of the optical pacing protocol for HF treatment, such as by the combination of pulse amplitude, pulse frequency, and pulse duration, is still needed. In addition, the VN is said to be composed of 20% efferent and 80% afferent fibers.^72,73^ The influence of afferent fibers in VNs also requires consideration to accomplish further development of VNS in HF.^74^

### Tragus nerve stimulation

Given that the auricular branch of the VN is connected to the skin of the tragus, stimulation at this site was thought to be viable as a method to stimulate the VN noninvasively.^75^ Stimulation of the tragus nerve preferably activates afferent rather than efferent vagal fibers, a phenomenon which appears to demonstrate a more significant inhibition of sympathetic activity.^75,76^ Also, this avoids additional stimulation of sympathetic fibers that may inadvertently be stimulated by the VNS.^75^ It was already shown that AF induced by rapid atrial pacing in canines and humans can be attenuated by tragus stimulation.^77,78^ In canines, this effect was observed to disappear following bilateral vagal transection.^77,78^ Another study found that patients receiving tragus stimulation for one hour daily for six months presented a 14-day continuous electrocardiogram atrial fibrillation burden that was 85% lower than in the sham earlobe stimulation group.^79^ Compliance with daily use was at a comparable rate to that in medication studies. In myocardial infarction–induced dogs, tragus stimulation after 90 days significantly reduced the infarct size by approximately 50%, together with reducing cardiac fibrosis and NE. The EF was significantly increased but the cardiac output was roughly unchanged.^80^ An ST-segment-elevation myocardial infarction study was conducted in which patients were randomly assigned to receive percutaneous coronary intervention and tragus stimulation or percutaneous coronary intervention and sham stimulation.^81^ The tragus and sham stimulations were initiated once the patient arrived in the catheterization room and lasted for two hours after balloon inflation. All patients were receiving guidelines-directed medical therapy and the results revealed markedly significant decreases in ventricular tachycardia, NT pro-BNP peptide level, and inflammatory markers in the tragus stimulation group. Also, the EF was significantly increased while the wall motion index was significantly decreased on echocardiography at just five to seven days after reperfusion, pointing to a possible application in HF.

These results are promising given that the therapy is self-administered, easily reversible, and contains essentially no significant risks. Tragus stimulation also possibly avoids the complications noted with cervical VNS such as dysphonia and coughing due to it being a noninvasive therapy.^75^ The issue with tragus stimulation for the treatment of HF, however, is that optimal dosing and stimulation parameters have not been determined to date.^75^ This is more an issue due to the lack of studies performed on testing differences in stimulation then related to problems with the therapy itself. There have also been disappointing outcomes among related studies on VNS in HF as mentioned above. Overall, the heterogenicity of HF combined with different VNS stimulation parameters used in the available studies points to the lack of an optimal VNS stimulation protocol for HF. However, despite this, some secondary outcomes did show significant improvements.^81^

### Cardiac contractility modulation

Cardiac contractility modulation (CCM) is accomplished by a biphasic impulse of 7.5 volts for 22 ms to the right ventricular septum during the absolute refractory period for five to 12 hours per day.^82^ CCM is used in NYHA class II or III patients with an EF of less than 35% and normal or mildly prolonged QRS duration who do not meet the criteria for cardiac resynchronization therapy (CRT).^82^ CCM increases septal contractility, which has been shown in rats to cause a reflex activation of vagal afferent fibers.^83,84^ This causes a reduction in excess sympathetic activation similar to that in CRT. Muscle sympathetic nerve activity decreased after three months of CCM in a patient with HF with reduced EF (HFrEF).^83^ Other mechanisms of action include attenuating myocardial fibrosis, increasing contractility via the upregulation of L-type calcium channels causing calcium uptake into the sarcoplasmic reticulum, and gene-remodeling to cause the more juvenile cardiomyocyte phenotype of HF to revert back to the normal adult cardiomyocyte.^83,84^

The Evaluate Safety and Efficacy of the OPTIMIZER System in Subjects with Moderate-to-severe HF (FIX-HF-4) study was a double-blinded, prospective, crossover study where HFrEF patients on optimized medical treatment (OMT) received 12 weeks of CCM therapy and showed an improvement in both their exercise tolerance and quality of life.^85^ The FIX-HF-5 study, a randomized longitudinal study of OMT versus OMT and CCM, found a safety analysis that was similar to CCM.^86^ Results for the primary endpoint, ventilatory anaerobic threshold, displayed no difference, while the secondary endpoints of NYHA class, normal mixed venous oxygen tension (pvO_2_), and Minnesota Living with Heart Failure Questionnaire (MLWHFQ) score experienced significant improvement.^86^ Details on ventilatory anaerobic threshold were required by the United States Food and Drug Administration due to the study being unblinded despite it not commonly being of relevance in later stages of HF.^82^ A confirmatory study, FIX-HF-5c, also reported significant improvements in pvO_2_, MLWHFQ score, NYHA class, and six-minute walk test results together with a reduction in cardiovascular death and HF hospitalization rate from 10.8% to 2.9% (p = 0.048).^87^ A meta-analysis of CCM revealed significant improvements in pvO_2_, six-minute walk test results, and MLWHFQ score.^88^

### Baroreceptor activation therapy

The carotid body and sinus are innervated by the PNS through vagus and glossopharyngeal fibers and the SNS via cervical sympathetic ganglia and contain a baroreceptor that encompasses both chemoreceptors and mechanoreceptors.^89^ Stimulation of carotid sinus mechanoreceptors results in afferent signals to the dorsal medulla, causing SNS attenuation and increased vagal tone, which leads to decreases in BP and HR.^89,90^ In HF patients, there is reduced sensitivity of the normal inhibitory function of the baroreceptor due to carotid body alterations via angiotensin II–mediated augmentation of carotid chemoreceptor sensitivity and central nervous system dysfunction, which are two of the mechanisms available for excessive sympathetic tone.^89^ Impairments in baroreceptor sensitivity in HF are associated with increased mortality rates.^91^ In canine HF models, baroreceptor activation therapy (BAT) decreased NR and angiotensin II levels, reversed LV remodeling, and improved survival.^92,93^

Efficacy and safety have already been shown for BAT therapy in hypertension. For HF, the BAROSTIM^®^ Hope for Heart Failure (HOPE4HF) phase II randomized controlled study noted improvements in six-minute walk test results, quality of life score, NYHA class, and NT pro-BNP level with no significant adverse events after six months.^94^ However, there was no significant difference in HF hospitalization or EF, possibly related to a placebo effect as the trials were not blinded.^94^ Recently, the Baroreflex Activation Therapy for HF (BeAT-HF) trial, a phase III multicenter randomized unblinded trial involving HF patients on OMT ineligible for CRT found that, after six months, BAT patients showed improved six-minute walk distances, MLWHFQ scores, and NT pro-BNP levels.^95^ This study lad to the provision of premarket approval by the Food and Drug Administration in 2019 for use of BAT therapy in HFrEF patients with NYHA classes II or III, an NT pro-BNP level of less than 1,600, or ineligibility for CRT. The postmarket phase will look at HF hospitalization and cardiovascular mortality rates.^95^ Nevertheless, the durability of treatment effects long-term and adverse effects years from implantation remain unknown.

### Spinal cord stimulation

Spinal cord stimulation (SCS) is performed by placing electrodes into the epidural space at the thoracic level and stimulating at 50 Hz with a duration of 0.2 ms.^96,97^ Stimulation will mitigate the signals that pass through the spinal cord—specifically, cardiac afferent through the dorsal root ganglia and preganglionic sympathetic efferent.^98^ The effects of SCS have been demonstrated by ischemic animal models, which suppressed ventricular arrhythmia and infarct sizes.^96,97^

Two clinical studies, the Determining the Feasibility of Spinal Cord Neuromodulation for the Treatment of Chronic HF (DEFEAT–HF) and the Spinal Cord Stimulation for HF (SCS HEART) trials, were performed to assess the utility of SCS in HF patients.^99,100^ Both studies were able to show safeness and feasibility; however, the SCS HEART study reported improvements in LV function and exercise tolerance, while the DEFEAT-HF trial did not. The main difference between the two studies was the stimulation areas and burden: the SCS HEART study stimulated at the T1-3 level continuously, while the DEFEAT-HF trial did so at the T2-4 level for 12 hours per day. The results indicate that the duty cycle, frequency, intensity of stimulation, and the area under stimulation can all affect the outcomes of SCS neuromodulation.

## Conclusion

Progression in the treatment of HF is ongoing and clinical data showing a significant improvement in treatment outcomes are constantly being published. Nevertheless, HF has remained a leading cause of death for decades. The cardiac autonomic nervous system and neurohormonal factors play important roles in cardiac function. Once an abnormality of the cardiovascular system occurs, an impact on disease development stemming from both the heart itself and the circumstances of the disease will occur. As summarized in **Table 1**, promising results from available studies suggest neuromodulation therapy is beneficial considering the potential side effects and complications. As a result, numerous trials are ongoing to assess the benefit of adding neuromodulation to HF management. The detection of pathological factors together with an optimal combination of pharmacological, device therapy, and invasive/noninvasive procedures can actively affect the progression of heart diseases from the early stages and may contribute to more significant improvements and better outcomes.

## Figures and Tables

**Table 1:** Results, Side Effects, and Complications from Neuromodulation Therapy

Neuromodulation Therapy	Principal Results from Studies	Potential Side Effects and Complications
CSD	Improved EF and 6MWT; suppressed HF progression	Horner’s syndrome, pneumothorax, hemothorax, hypotension, harlequin unilateral facial flushing, and hyperhidrosis
RND	Improved EF, 6MWT, NYHA class; decreased NT pro-BNP	Renal artery stenosis, renal artery dissection, UTI, and femoral pseudoaneurysms
VNS	Inconclusive; ANTHEM-HF reported improvements in EF, NYHA class, 6MWT, and MLWHFQ score, while INOVATE-HF and NECTAR-HF did not	Dysphonia, coughing, oropharyngeal pain, vocal cord paralysis, and lead fracture and malfunction
TNS	Reduced AF burden; in acute STEMI, EF increased, while VT, wall motion index, NT pro-BNP decreased	Electrode malfunction
CCM	Reduced HF hospitalization and CV death; improved pVO_2_, 6MWT, MLWHFQ score, NYHA class	Lead fracture, infection, and pericardial effusion
BAT	Improved 6MWT and MLWHFQ score; decreased NT pro-BNP	Localized numbness, dysphonia, and dysphagia
Spinal cord stimulation	Inconclusive; SCS HEART reported improved LV function and exercise tolerance, while DEFEAT-HD did not	Lead fracture, lead fracture, implant site hematoma, neck or back pain, and postprocedure decompensated HF

BAT: baroreceptor activation therapy; CCM: cardiac contractility modulation; CSD: cardiac sympathetic denervation; EF: ejection fraction; HF: heart failure; NYHA: New York Heart Association; NT-proBNP: N-terminal prohormone B-type natriuretic peptide: RND: renal nerve denervation: SCS: spinal cord stimulation; TNS: tragus nerve stimulation; UTI: urinary tract infection; VNS: vagus nerve stimulation.
